# Cost‐effectiveness of ipilimumab versus high‐dose interferon as an adjuvant therapy in resected high‐risk melanoma

**DOI:** 10.1002/cam4.4194

**Published:** 2021-08-17

**Authors:** Mia Salans, Patrick Travis Courtney, Anthony Yip, James D. Murphy

**Affiliations:** ^1^ University of California San Diego School of Medicine La Jolla California USA; ^2^ Department of Radiation Medicine and Applied Sciences University of California San Diego La Jolla California USA

**Keywords:** cost‐effectiveness, high‐dose interferon, immunotherapy, ipilimumab, melanoma

## Abstract

**Background:**

Adjuvant ipilimumab was found to improve the overall survival and reduce toxicity compared to high‐dose interferon (HDI) in patients with resected, high‐risk melanoma. However, the cost of ipilimumab is substantially higher than HDI. This study evaluates the cost‐effectiveness of ipilimumab as an adjuvant treatment in melanoma from a healthcare perspective.

**Methods:**

We designed a Markov model simulating resected, high‐risk melanoma patients receiving either ipilimumab or HDI. Transition probabilities, including risks of survival, disease progression, and toxicity, were ascertained from clinical trial data. Costs and quality of life measurements (health utilities) were extracted from the literature. Incremental cost‐effectiveness ratios (ICERs), defined as incremental costs divided by incremental quality‐adjusted life‐years (QALYs), assessed cost‐effectiveness. ICERs <$100,000/QALY were deemed cost‐effective. We measured model uncertainty with one‐way and probabilistic sensitivity analyses.

**Results:**

In our base case model, ipilimumab increased costs by $107,100 and increased effectiveness by 0.43 QALY, yielding an ICER of $392,600/QALY. Our model was moderately sensitive to the costs of ipilimumab, though the cost of ipilimumab would need to decrease by 44% for ipilimumab to become cost‐effective compared to HDI. The model was not sensitive to survival, toxicity, or other costs. Probabilistic sensitivity analysis showed that HDI would remain the cost‐effective treatment option 96.2% of the time at a willingness‐to‐pay threshold of $100,000/QALY.

**Conclusions:**

Adjuvant ipilimumab increases the survival and decreases the toxicity compared to HDI in resected, high‐risk melanoma patients, though this would not be considered cost‐effective due to the high price of ipilimumab.

## Graphical abstract

Ipilimumab is not a cost‐effective adjuvant treatment option compared with high‐dose interferon for resected, high‐risk melanoma. The cost of ipilimumab would have to decrease substantially to reach cost‐effectiveness.

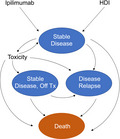

## 1 INTRODUCTION

With an estimated 96,480 new cases diagnosed in 2019, melanoma represents the fifth most common cancer in the United States (US).^1^ High‐risk melanoma comprises around 13% of new melanoma cases^2^ and is currently treated with resection followed by adjuvant systemic therapy to reduce the risk of relapse.^3^ Until recently, high‐dose interferon alfa‐2b (HDI) was the standard adjuvant treatment for high‐risk disease, but it has since been replaced by immune checkpoint inhibitors and targeted therapy.^3^ Low‐dose ipilimumab, a cytotoxic T‐lymphocyte‐associated protein 4 inhibitor, was recently found to improve the survival with reduced toxicity compared to HDI in the North American Intergroup E1609 trial among patients with resected, high‐risk melanoma.^4^ As a result, low‐dose ipilimumab now represents a standard adjuvant treatment option according to the current consensus guidelines.^3^

Despite the survival benefit associated with ipilimumab, it is far more expensive than the prior standard of care, HDI, reaching close to $70,000 for one course of treatment.^5^ The incidence of melanoma has more than doubled since 1985,^2^ further emphasizing the importance of considering cost in this patient population. This study evaluated the cost‐effectiveness of ipilimumab in comparison with HDI for patients with resected, high‐risk melanoma. Through this investigation, we aimed to compare the value of these treatments while taking into account differences in survival, cost, and quality of life.

## 2 METHODS

### 2.1 Decision model

**FIGURE 1:**
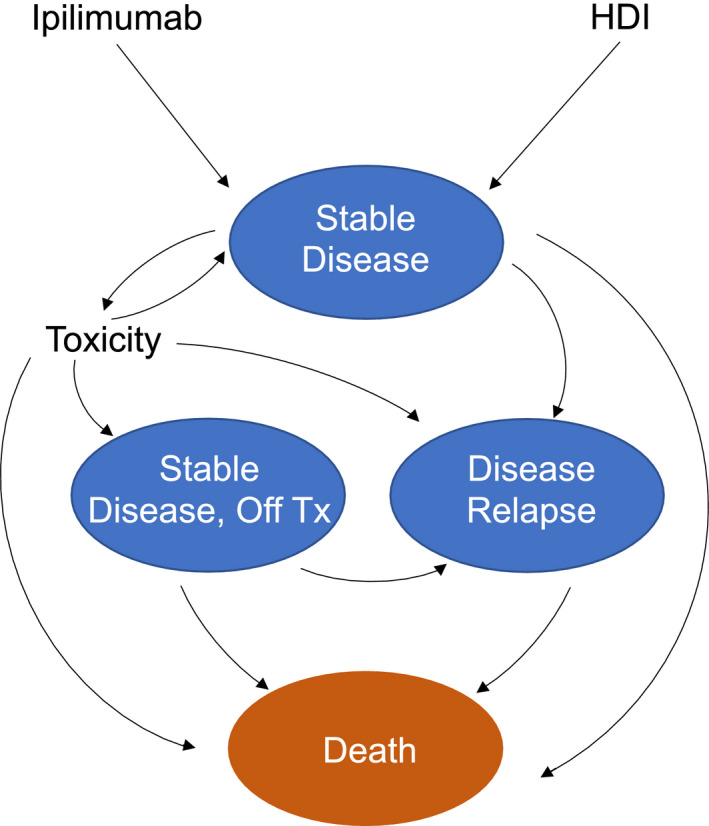
Transition state diagram. This figure shows the potential health states patients could experience in this analysis. Ovals represent distinct disease states and arrows represent potential transitions between different disease states. HDI, high‐dose interferon; Tx, treatment

Using TreeAge Pro Healthcare,^6^ we constructed a Markov model to compare the cost‐effectiveness of ipilimumab and HDI among patients with resected high‐risk melanoma (stages IIIB, IIIC, M1a, or M1b). Our model integrated survival, toxicities, costs, and patient quality of life, incorporating data whenever possible from the E1609 randomized phase III trial.^4^ E1609 randomized patients to HDI or one of two doses of ipilimumab (3 mg/kg or 10 mg/kg). In this trial, the high‐dose ipilimumab (10 mg/kg) cohort experienced more toxicity than the 3 mg/kg group without a significant improvement in survival compared to HDI. Therefore, this study focused on the ipilimumab cohort receiving doses of 3 mg/kg. The four main health states in our model included “stable disease on treatment,” “stable disease off treatment,” “disease relapse,” and “death.” Patients moved through the Markov model following the state transition diagram (Figure 1). Patients incurred a health utility deduction within the “stable disease on treatment” health state if they experienced toxicity. Patients who discontinued the study treatment regimen due to a drug‐related adverse event incurred a health utility deduction and moved to the “stable disease off treatment” health state. Patients experiencing disease relapse within the “stable disease on treatment” or “stable disease off treatment” states moved to the “disease relapse” health state. We employed a 1‐month cycle length over a 10‐year time horizon and implemented a half‐cycle correction to improve survival estimation. This cost‐effectiveness analysis follows standard design and reporting guidelines published elsewhere.^7^

### 2.2 Treatment details

Following the E1609 trial protocol, patients were treated with 3 mg/kg ipilimumab administered intravenously (IV) every 3 weeks for four doses (induction), followed by the same dose every 12 weeks for up to four additional doses (maintenance). Patients in the HDI group received 20 million units/m^2^ IV HDI per day, 5 days per week, for 4 weeks (induction), followed by 10 million units/m^2^ per day subcutaneously every other day, 3 days per week, for 48 weeks (maintenance).^4^ Ipilimumab and HDI doses were calculated using average weight (84.2 kg) and body surface area (1.79 m^2^), among American adults.^8^ Treatment was continued for a maximum of 60 and 52 weeks in the ipilimumab and HDI groups, respectively, or until disease progression, unacceptable toxicity, or withdrawal of consent.

### 2.3 Model probabilities

**FIGURE 2:**
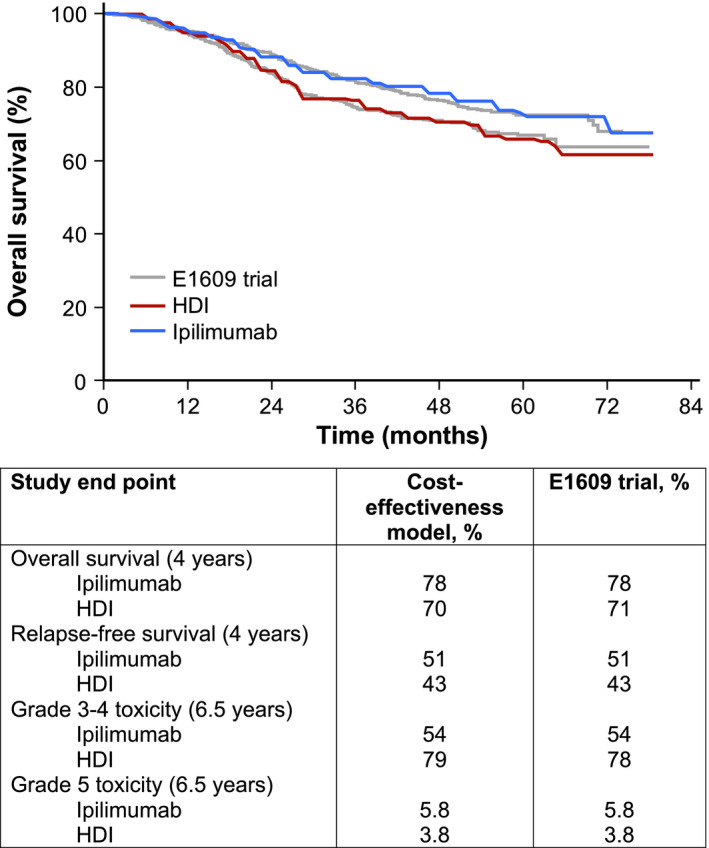
Survival and toxicity validation. This figure shows the cost‐effectiveness model validation results. The top panel shows how our model predicts the overall survival compared with the E1609 trial. The bottom panel shows how our model predicts the overall survival, relapse‐free survival, grade 3–4 toxicity, and grade 5 toxicity compared with the E1609 trial

All patients entered the model in the “stable disease on treatment” health state and received either ipilimumab or HDI. Following treatment initiation, patients could experience a toxicity, melanoma relapse, or death. Similar to other analyses,^9^ we considered only grade 3–5 toxicity in our analysis. Patients who experienced unacceptable levels of toxicity but did not relapse moved to the “stable disease off treatment” state. Probabilities of toxicity, relapse, and death were derived from the E1609 trial and were validated against the trial results (Figure 2). We calculated monthly probabilities of melanoma relapse and death to simulate the Kaplan–Meier overall survival (Figure 2) and progression‐free survival (not shown) curves provided in the study. The E1609 trial reported survival through 78 months following treatment initiation. Our base case analysis assumed that melanoma‐specific monthly mortality rates would follow population estimates derived from the Surveillance, Epidemiology, and End Results (SEER) databases beyond 78 months.^10^ We varied this long‐term survival assumption in a sensitivity analysis, in which we assumed a more optimistic scenario where patients alive beyond 78 months were cured of melanoma, and the risk of death followed age‐adjusted mortality probabilities retrieved from the US Social Security Death Index.^11^ Additionally, we varied our model time horizon in a sensitivity analysis to determine the cost‐effectiveness of ipilimumab versus HDI over a lifetime horizon.

### 2.4 Costs

Our base case analysis incorporated costs from a healthcare system perspective. We also performed a sensitivity analysis to examine the cost‐effectiveness from a societal perspective, considering patient time, transportation, lost productivity, and caregiver costs. The costs of ipilimumab and HDI were determined by subtracting 7% from each drug's average wholesale price^9^, ^12^ and adding infusion costs. Costs of treatment‐related toxicities, as well as costs of patient time and productivity loss, caregivers, and parking, meals, and travel were derived from the literature^13^, ^14^, ^15^, ^16^, ^17^, ^18^, ^19^, ^20^ (Table 1). Costs of toxicity were calculated as a weighted average of the most frequent grade 3+ toxicities occurring among patients in the E1609 trial (Table S1), excluding toxicities that occurred in <3% of patients or were primarily laboratory value abnormalities (e.g., increased lipase or liver enzymes). Patients who relapsed incurred the average estimated cost of melanoma relapse.^21^ Palliative care costs were incurred by all patients who died in the month prior to death.^21^ All medical costs, including drug, toxicity, and disease management costs, were adjusted to 2020 US dollars using the medical care component of the consumer price index^22^; societal costs were adjusted to 2020 US dollars using the total consumer price index.^22^

**TABLE 1:** Parameters for base case cost‐effectiveness model

Parameter	Value (95% CI)	Distribution	Source
Patient age	54	Gamma	Tarhini 2019
Costs (in 2020 USD)^a^			
Drug costs (per cycle)^b^			
Ipilimumab (per infusion)^c^	54,850 (33,349–76,351)	Gamma	UpToDate
HDI induction^c^	26,750 (16,264–37,236)	Gamma	UpToDate
HDI maintenance^c^	7,782 (4,731–10,833)	Gamma	UpToDate
Infusion costs (per cycle)			
Ipilimumab induction	196 (119–273)	Gamma	Tringale 2018
Ipilimumab maintenance	49 (30–68)	Gamma	Tringale 2018
HDI induction	2,954 (1,796–4,112)	Gamma	Tringale 2018
HDI maintenance	1,772 (1,077–2,467)	Gamma	Tringale 2018
Drug toxicity costs (per cycle)^d^			
Ipilimumab	7,975 (4,849–11,101)	Gamma	Barzey 2013, Bohensky 2016
HDI	1,625 (988–2,262)	Gamma	Barzey 2013, Simon 2001
Disease costs (per cycle)			
Stable disease^e^	1,066 (571–1,307)	Gamma	Barzey 2013
Progressed disease	11,365 (6,910–15,820)	Gamma	Hillner 1997
Palliative care and death (one‐time cost)	22,731 (13,820–31,642)	Gamma	Hillner 1997
Societal costs (per cycle)			
Patient time/salary loss			Bureau of Labor Statistics
Ipilimumab induction	204 (124–284)	Gamma	
Ipilimumab maintenance	51 (31–71)	Gamma	
HDI induction	3,062 (1862–4262)	Gamma	
HDI maintenance	1837 (1117–2557)	Gamma	
Stable disease off treatment	153 (93–213)	Gamma	
Parking, meals, and travel			Lauzier 2011
Ipilimumab induction	65 (40–90)	Gamma	
Ipilimumab maintenance	16 (10–22)	Gamma	
HDI induction	980 (596–1364)	Gamma	
HDI maintenance	588 (358–818)	Gamma	
Stable disease off treatment	49 (30–68)	Gamma	
Caregiver	619 (401–882)	Gamma	Li 2013
Health utilities (per year)			
Stable disease on treatment	0.83 (0.50–1.00)	Beta	Wang 2017
Stable disease off treatment	0.96 (0.58–1.00)	Beta	Cormier 2007
Relapsed disease	0.52 (0.44–0.61)	Beta	Kohn 2017
Drug toxicity disutility^f^			
Ipilimumab	0.0134 (0.0081–0.0187)	Beta	Kohn 2017
HDI	0.0126 (0.0076–0.0175)	Beta	Kohn 2017, Hall 2019, Matza 2015
Death	0		

Abbreviations: CI, confidence interval; HDI, high‐dose interferon; USD, United States Dollar.

^a^ Costs adjusted for inflation when appropriate.

^b^ Average wholesale price with 7% reduction

^c^ Dose per cycle calculated using average weight (ipilimumab) and body surface area (HDI) for American adults.

^d^ Calculated as average cost of toxicity using weighted frequencies of grade 3–4 treatment‐related adverse events for each treatment arm in the E1609 trial. Costs for individual toxicities were derived from the literature and include all care required to manage each toxicity. References and individual toxicity costs are summarized in Table S1.

^e^ Includes cost in 2020 USD of monitoring and management of advanced melanoma patients on ipilimumab.

^f^ Calculated as average disutility of toxicity using weighted frequencies of grade 3–4 treatment‐related adverse events for each treatment arm in the E1609 trial. Disutilities for individual toxicities were derived from the literature. References and individual toxicity disutilities are summarized in Table S2.

### 2.5 Outcome measures

Quality‐adjusted life‐years (QALYs) measured effectiveness. QALYs express the product of health utility over time. Health utility evaluates health‐related quality of life with a range between 0 (death) and 1 (optimal health).^7^ Health utility values corresponding to different health states were obtained from the literature.^23^, ^24^, ^25^ Health utility decreased with disease progression or after experiencing treatment‐related toxicity (Table 1). The health utility decrements (health utility tolls) associated with toxicity were calculated as a weighted average of grade 3–4 toxicity events in the E1609 trial (see Table S2 for the most frequently occurring grade 3–4 treatment‐related toxicities in the trial and their associated disutilities). A 3% annual discount rate was used for all costs and QALYs.^9^, ^12^

### 2.6 Analysis

An incremental cost‐effectiveness ratio (ICER) measured the cost‐effectiveness of ipilimumab versus HDI. ICERs represent the difference in cost between two therapies divided by their difference in QALYs. Treatments with an ICER below a willingness‐to‐pay threshold of $100,000/QALY were deemed cost‐effective, with reported values rounded to the nearest $100. We performed one‐way sensitivity analyses on all variables included in the model to determine which factors affected cost‐effectiveness. We conducted a probabilistic sensitivity analysis using a Monte Carlo simulation with 100,000 iterations to examine the effect of uncertainty in transition probabilities, costs, and health utilities on our model. Gamma distributions modeled cost estimates, while beta distributions modeled transition probabilities and health utilities. Uncertainty in disease progression and death was modeled to reflect the hazard ratios and confidence intervals reported in the E1609 trial.^4^ The standard deviations of cost, health utilities, and probability of toxicity were ascertained from the literature when possible, and unknown standard deviations were assumed to be 20% of the mean.^9^, ^12^ We varied the unknown standard deviation values from 10% to 40% of the mean in a sensitivity analysis, which had no effect on our analyses (results not shown).

## 3 RESULTS

### 3.1 Base case analysis

In our base case analysis, ipilimumab raised the total cost of treatment by $170,100 from $461,000 with HDI to $631,100 with ipilimumab. Effectiveness improved by 0.43 QALYs with ipilimumab, from 5.00 QALYs with HDI to 5.43 QALYs with ipilimumab. The ICER for ipilimumab compared with HDI was $392,600/QALY. Adjuvant ipilimumab would therefore not be considered cost‐effective for resected, high‐risk melanoma at a willingness‐to‐pay threshold of $100,000/QALY. Our cost‐effectiveness analysis from a societal perspective yielded a modest reduction in the ICER of ipilimumab versus HDI to $346,200/QALY.

### 3.2 One‐way sensitivity analysis

**FIGURE 3:**
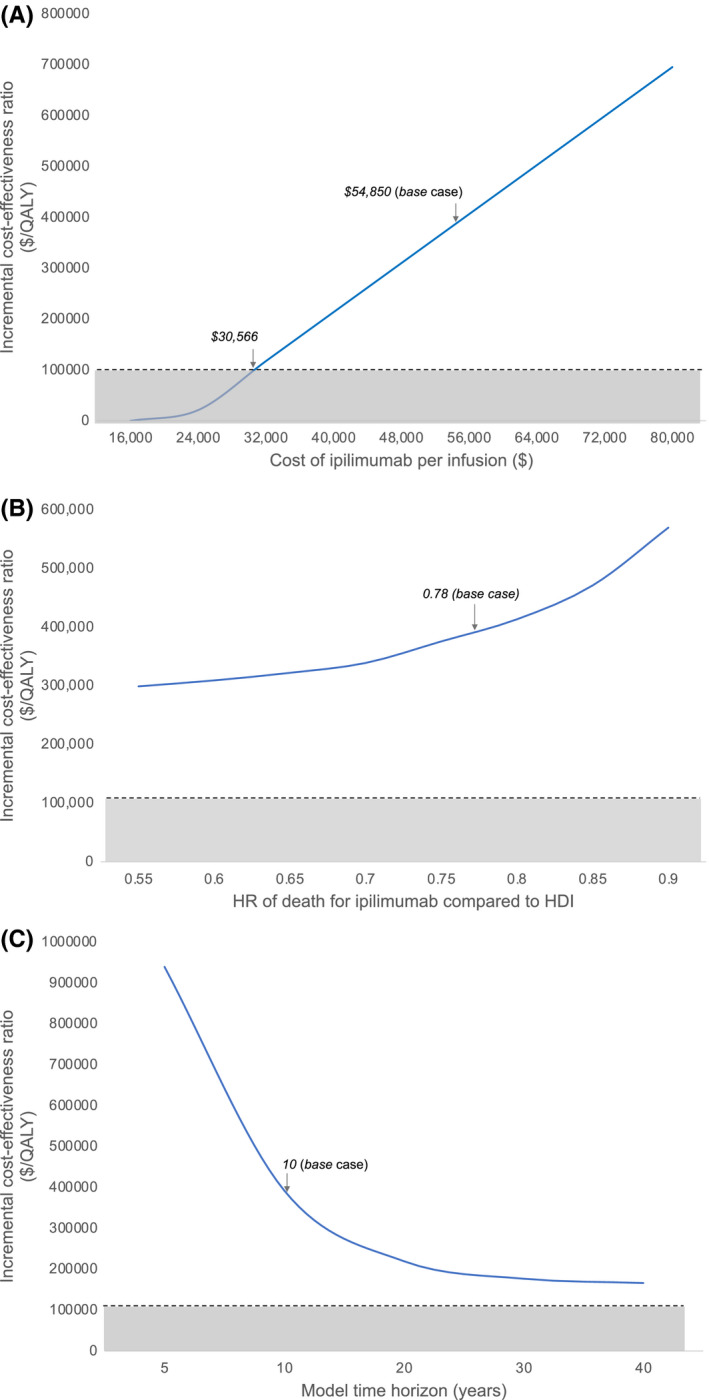
One‐way sensitivity analyses. These graphs represent the cost‐effectiveness of ipilimumab compared to HDI, measured by incremental cost‐effectiveness ratios (ICERs). The horizontal dashed line represents the willingness‐to‐pay threshold, which is set at $100,000/QALY, and the region below the dashed line represents ICERs at which ipilimumab would be considered cost‐effective compared to HDI. Panel A illustrates how the ICER changes with varying costs of ipilimumab per infusion. Panel B demonstrates how the ICER varies with the hazard ratio of death for ipilimumab compared to HDI. Panel C demonstrates how the ICER varies with model time horizon. HDI, high‐dose interferon; HR, hazard ratio; QALY, quality‐adjusted life‐year

Our model was most sensitive to ipilimumab's cost. The cost of an ipilimumab infusion would need to fall by 44%, or from $54,850 to $30,566, for ipilimumab to become cost‐effective (Figure 3A) at a willingness‐to‐pay threshold of $100,000/QALY. The ICERs for ipilimumab versus HDI when ipilimumab's cost was reduced to 75% and 50% of its current cost were $227,400/QALY and $62,300/QALY, respectively. Our model was also somewhat sensitive to the time horizon used in our analysis. Ipilimumab approached cost‐effectiveness when we employed a time horizon of 40 years, with an ICER of $166,300/QALY (Figure 3B). Our analysis was minimally sensitive to the proportion of patients with stable disease that discontinued ipilimumab before completing the full treatment regimen. Per the E1609 trial, our base case analysis assumed that 39.1% of patients discontinued ipilimumab prior to the 60‐week maximum. Increasing the percentage of individuals who did not complete the full course to 60% led to an ICER of $323,500/QALY, still above the threshold of being considered cost‐effective.

Our model was insensitive to other variables, including toxicity, rate of disease progression, health utilities, and costs other than that of ipilimumab. Our model was also not especially sensitive to survival assumptions. Patients in the E1609 trial taking ipilimumab had a 22% lower risk of death versus patients treated with HDI (hazard ratio [HR] = 0.78).^4^ The ICER fell to $299,000/QALY if we assumed that ipilimumab reduced the risk of death by 45% (HR = 0.56, below the lower end of the 95% CI for the HR reported in the E1609 trial) (Figure 3C). In our base case analysis, the probability of death beyond the 78 months reported in the E1609 trial followed SEER data; however, if we assumed that patients still living beyond 78 months were cured of their disease, ipilimumab's ICER was reduced to $388,000/QALY. These sensitivity analyses are summarized in Table S3.

### 3.3 Probabilistic sensitivity analysis

**FIGURE 4:**
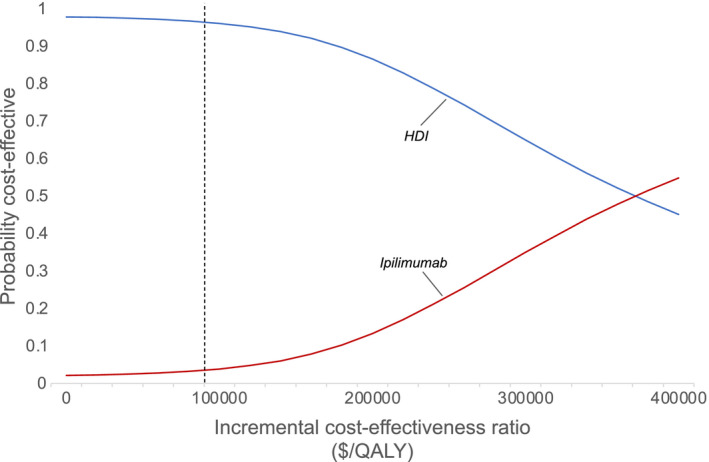
Probabilistic sensitivity analysis. This plot shows the results of a probabilistic sensitivity analysis comparing the cost‐effectiveness of ipilimumab with HDI for resected, high‐risk melanoma. The dashed line represents a wiliness‐to‐pay threshold of $100,000/QALY. HDI, high‐dose interferon; QALY, quality‐adjusted life‐year

**FIGURE 5:**
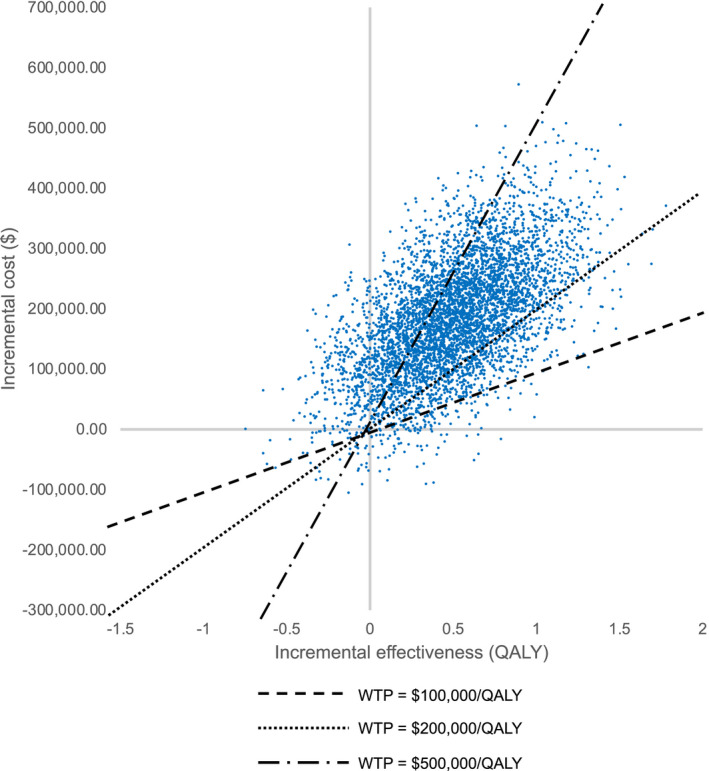
Probabilistic sensitivity analysis scatter plot. This plot shows the distribution of incremental costs and incremental effectiveness of individual iterations of the probabilistic sensitivity analysis comparing the cost‐effectiveness of ipilimumab versus HDI. The dashed lines represent willingness‐to‐pay thresholds of $100,000/QALY, $200,000/QALY, and $500,000/QALY (see figure legend). Points on the graph represent individual iterations of the probabilistic sensitivity analysis. Points to the right of each dashed line indicate iterations in which ipilimumab was cost‐effective compared with HDI at that specific willingness‐to‐pay threshold; points to the left of each dashed line indicate iterations in which ipilimumab was cost‐ineffective compared to HDI at that specific willingness‐to‐pay threshold. WTP, willingness‐to‐pay, QALY, quality‐adjusted life‐year

The impact of variability in costs, toxicity, health utility, and survival was assessed simultaneously in a probabilistic sensitivity analysis over 100,000 iterations. This analysis illustrated that our model's results were relatively robust. In comparison with ipilimumab, HDI would be cost‐effective 96.2% of the time at a willingness‐to‐pay threshold of $100,000/QALY (Figure 4). HDI continued to represent the cost‐effective treatment 86.7% of the time at a willingness‐to‐pay threshold of $200,000/QALY. Figure 5 demonstrates a scatterplot of the incremental costs versus incremental effectiveness of ipilimumab compared with HDI at various willingness‐to‐pay thresholds from individual iterations in the probabilistic sensitivity analysis.

## 4 DISCUSSION

The epidemiologic and therapeutic landscapes of melanoma have substantially shifted over the past several decades. Specifically, the rising incidence of melanoma coupled with the advent of novel immunotherapeutic agents has resulted in a dramatic increase in the number of melanoma survivors. The standard of care for resected, high‐risk melanoma now consists of an array of novel therapeutics, including immune checkpoint inhibitors such as ipilimumab, nivolumab, pembrolizumab, or dabrafenib/trametinib among individuals with a BRAF V600 activating mutation,^3^ all of which are significantly more expensive than the previous generation of treatments.^5^, ^26^ As such, it is crucial to understand the value of these therapies in comparison with the previous standard of care, particularly as they become more widely used. The E1609 trial found that ipilimumab was superior to HDI with regards to both efficacy and toxicity^4^ in resected, high‐risk melanoma. Despite improved outcomes among patients receiving ipilimumab, we found this treatment would unlikely be considered cost‐effective at current willingness‐to‐pay thresholds.

This is the first study to our knowledge to assess the cost‐effectiveness of any immune checkpoint inhibitor as an adjuvant therapy for resected, high‐risk melanoma compared to HDI. Prior studies have found ipilimumab to be cost‐ineffective in comparison with other immunotherapeutic agents, including pembrolizumab^27^ and nivolumab,^28^ in patients with metastatic or unresectable melanoma. Yet, patients with resectable melanoma comprise a distinct population with longer survival than patients with advanced melanoma.^29^ Longer survival allows for greater accumulation of QALYs, which could in theory increase the likelihood that therapies will be cost‐effective. Despite the differences between resectable and advanced melanoma, the findings of this study demonstrate that ipilimumab is not a cost‐effective adjuvant treatment option in the setting of high‐risk, resected melanoma.

The impact of the cost of ipilimumab on cost‐effectiveness represents a key finding in our analysis. While many factors contribute to ipilimumab treatment costs, the cost of ipilimumab therapy is primarily influenced by drug costs and duration of treatment. Our model demonstrates that ipilimumab would only become cost‐effective after a substantial reduction in cost. With respect to treatment duration, the optimal length of adjuvant treatment for melanoma has not yet been established^30^; however, metastatic melanoma patients treated with longer courses of dual checkpoint inhibitors (pembrolizumab/nivolumab) may have a reduced risk of progression compared to those treated with shorter durations.^31^ Future studies will likely shape the optimal treatment duration of immunotherapy, and we will need additional research to assess the value of these agents in resectable melanoma.

Importantly, the ICER for ipilimumab approached $100,000/QALY when we performed our analysis over a lifetime horizon, suggesting that ipilimumab could eventually represent a cost‐effective treatment option among melanoma patients surviving at least 40 years beyond their initial diagnosis. Similar findings are often seen in cost‐effectiveness analyses of interventions associated with high upfront costs and longer term health benefits.^32^ Our base case model likely captured a greater proportion of ipilimumab's costs than benefits, yielding a higher ICER for ipilimumab. Of note, the E1609 trial only reported survival data through 7 years after treatment initiation; thus, we estimated survival probabilities beyond the trial length in our model using SEER data. Prospective clinical trials that assess survival over longer periods of time are needed to better characterize the long‐term costs and quality of life associated with ipilimumab. Nevertheless, our findings demonstrate ipilimumab's potential to become a cost‐effective treatment option in the long‐term.

Our model was not sensitive to survival. Indeed, the ICER plateaued above $100,000/QALY when examining a variety of assumptions regarding the survival efficacy of ipilimumab compared to HDI. Even with substantial improvements in survival with ipilimumab, the ICER did not cross the threshold where ipilimumab would be considered cost‐effective compared to HDI. Additionally, the ICER did not substantially decrease when we assumed that all patients still living beyond 78 months were cured of melanoma. This somewhat paradoxical finding comes from the reality of improved survival—while longer survival leads to gains in QALYs, these come with additional costs, including costs of follow‐up and disease monitoring that offset any QALY gains and preclude ipilimumab from becoming cost‐effective by today's standards. Our findings support other research evaluating cancer therapeutics in the modern era where cost‐effectiveness does not depend on survival.^9^, ^33^

This study has limitations worth mentioning. We derived model inputs primarily from a single randomized trial (E1609). Incorporating data from a randomized trial represents a strength of our analysis,^34^ though ideally we would include more robust, long‐term data for the cohort treated with ipilimumab. While our model was insensitive to various survival assumptions, it would still benefit from the inclusion of additional clinical trial data to increase generalizability of our findings. Another limitation relates to the fact we relied on the literature for model inputs for cost and health utilities. While variation in these estimates could impact our results, the cost‐effectiveness model outcomes did not depend on these inputs, which suggests that incorporating more precise model estimates would unlikely substantially change our results or conclusions. Of note, with the relative novelty of immunotherapy, we do not have a clear understanding of the long‐term costs and survival associated with these therapies. Any substantial variation in costs or outcomes could influence our assessment of cost‐effectiveness. Prospective longitudinal assessment of costs and quality of life would help to improve the estimations of cost‐effectiveness and should be integrated into future research involving high‐cost therapies.

As the incidence of melanoma continues to rise, it is crucial to understand and address major cost drivers in this patient population. This may help to facilitate the development of effective cost containment strategies focused on reducing financial burden on patients and the healthcare system as a whole. Despite improved survival and reduced toxicity, ipilimumab is not a cost‐effective adjuvant therapy for patients with resected, high‐risk melanoma. Nevertheless, our findings demonstrate ipilimumab has the potential to become cost‐effective with reductions in cost and treatment duration, rather than improved efficacy.

## 5 ETHICS STATEMENT

This study was exempt from review by the UC San Diego institutional review board as it involved the analysis of previously published data.

## CONFLICT OF INTEREST

JDM receives compensation for consulting from Boston Consulting Group for work unrelated to this project.

## Supporting information

Table S1

Table S2

Table S3

## Data Availability

The data that support the findings of this study are available from the corresponding author upon reasonable request.
