# Supplementary material for: Cost‐effectiveness of ipilimumab versus high‐dose interferon as an adjuvant therapy in resected high‐risk melanoma
Source: Cancer Med. 2021 Aug 17;10(19):6618–26. doi: 10.1002/cam4.4194 (PMC8495287; doi:10.1002/cam4.4194)
Supplement: Supplementary file 1 — Table S1 [file CAM4-10-6618-s003.docx]

**Supplementary Table 1. Associated costs of grade 3-4 treatment-related toxicities**

| **Toxicity^a^** | **No. of patients (%)^b^** | **Costs in 2020 USD^c^** | **Reference** |
| --- | --- | --- | --- |
| **Ipilimumab** |  |  |  |
| Diarrhea/colitis | 67 (13.0) | 11,106 | Barzey 2013 |
| Rash | 24 (4.7) | 173 | Bohensky 2016 |
| Hypophysitis/hypopituitarism | 17 (3.3) | 6457 | Barzey 2013 |
| *Weighted average*^d^ | - | *7975* |  |
| **High Dose Interferon** |  |  |  |
| Myelosuppression | 206 (39.6) | 1108 | Barzey 2013 |
| Fatigue | 120 (23.1) | 2683 | Barzey 2013 |
| Nausea/vomiting | 37 (7.1) | 1870 | Barzey 2013 |
| Depression/anxiety | 24 (4.6) | 514.28 | Simon 2001 |
| *Weighted average*^†^ | *-* | *1625* |  |

^a^Refers to grade 3-4 treatment-related toxicities that occurred in ≥3% of patients and were not primarily laboratory value abnormalities (e.g., increased lipase or liver enzymes) in that specific treatment arm in the E1609 trial.

^b^Percentage within treatment arm: Ipilimumab (N=516), High Dose Interferon (N=520).

^c^Cost per one-month cycle.

^d^Calculated as an average cost of toxicity using the weighted frequency of occurrence. This value was used in the base case model.
