# Supplementary material for: Cost‐effectiveness of ipilimumab versus high‐dose interferon as an adjuvant therapy in resected high‐risk melanoma
Source: Cancer Med. 2021 Aug 17;10(19):6618–26. doi: 10.1002/cam4.4194 (PMC8495287; doi:10.1002/cam4.4194)
Supplement: Supplementary file 3 — Table S3 [file CAM4-10-6618-s001.docx]

**Supplementary Table 3. ICERs for various model assumptions**

| **Model** | **ICER ($/QALY)** |
| --- | --- |
| Base case  **Perspective**  Health care payer  Societal  **Survival Assumptions Beyond Trial Range^a^**  Patients alive at 78 months follow SEER survival data for high-risk melanoma  Patients alive at 78 months cured of disease^b^  **Model Time Horizon**  10 years  40 years | 392,600  392,600  346,200  392,600  388,000  392,600  166,300 |

Abbreviations: QALY = quality-adjusted life year, SEER = Surveillance, Epidemiology, and End Results

^a^E1609 trial reported survival data through 78 months.

^b^Survival followed normal age-adjusted mortality rate obtained from the US Social Security Death Index.
